# Longitudinal studies on interdisciplinary teaching models incorporating plastic surgery

**DOI:** 10.1308/rcsann.2025.0006

**Published:** 2025-04-08

**Authors:** N Sivathasan, B Robertson

**Affiliations:** ^1^ American Academy of Cosmetic Surgery, Chicago, USA; ^2^ NHS Tayside, UK

**Comment on**

H Bhachoo, SC Glossop, LR Mattey *et al*. Undergraduate deficits in plastic surgery exposure and awareness of the specialty: a systematic review. *Ann R Coll Surg Engl* 2025; **107**: 12–17

The systematic review (of ten papers published between 2010 and 2021) by Bhachoo *et al* (2025) has, once more, highlighted the deficits in undergraduate medical curricula regarding exposure to plastic surgery.^1^ Bhachoo *et al* touched upon the resultant challenges for subsequent interest in the specialty, and emphasised the importance of early and structured educational exposure. However, we felt their themes were rather desultory, and they provided no pragmatic solutions, particularly with reference to patient-centred outcomes.

In 2018, we published empirical evidence of the consequences of inadequate exposure to plastic surgery.^2^ Although the comparable themes in both papers pertain to educational value (plastic surgery in medical curricula), our study quantitatively demonstrated that doctors in training who experienced plastic surgery rotations during their undergraduate training performed significantly better in identifying and managing clinical conditions pertinent to this specialty; and these findings, in terms of enhanced patient-safety, underscored the argument for mandatory inclusion of plastic surgery in medical curricula, which we had opined should be sustained and integrated exposure.

Many years on, we still feel one-day courses in isolation are inadequate, particularly when discretionary and often only attended by those already with an interest in the specialty, as promoted by Bhachoo *et al*. In addition, continuing societal biases against cosmetic (plastic) surgery contribute to misconceptions among medical students and doctors in training about the closely related, but still different, specialty of reconstructive (plastic) surgery – and such would adversely affect attendance at optional courses. The aim is to increase the extent of educational exposure; hence, Robertson *et al* additionally provided an actionable solution: curricular integration into teaching modules with related specialties like dermatology and emergency medicine to contextualise reconstructive (plastic) surgery into broader medical practice. We had provided the evidence (59% vs 18%, *p* = 0.0001) to support the consequential improvement in clinical referrals by doctors who had been exposed to the specialty compared with those who had not.

By combining Bhachoo *et al*’s systematic review with Robertson *et al*’s empirical findings, a compelling case is made for structured, widespread and contextually integrated plastic surgery education. However, we feel, this is already established. Accordingly, we suggest that any future studies are longitudinal in nature, and involve medical schools with interdisciplinary teaching models that incorporate plastic surgery to assess the evidence-based impact on the following: (1) patient safety, (2) evolution of this specialty in the medical workforce and (3) multidisciplinary collaboration.
